# Supplementary figures and images for: Counting is almost all you need
Source: Front Immunol. 2023 Jan 20;13:1031011. doi: 10.3389/fimmu.2022.1031011 (PMC9896581; doi:10.3389/fimmu.2022.1031011)

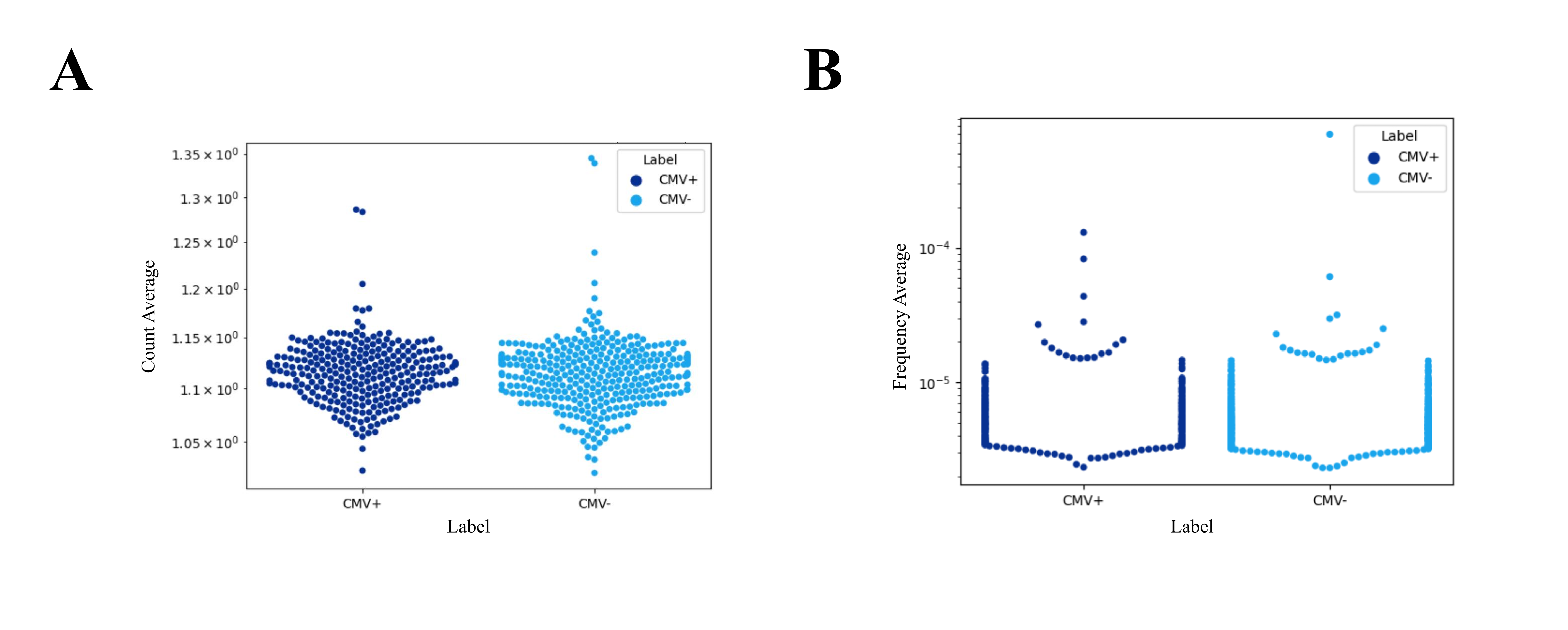

Supplement: Supplementary Image 1 — (A) A swarm plot of the different repertoires in the data. Each dot represents a repertoire. The y-axis represents the average count of a TCR in a repertoire, where the count of a TCR is defined as the number of clones the TCR has in the repertoire. It is clear that there is not a big difference in the count distribution between positive and negative repertoires. (B) A swarm plot of the different repertoires in the data. Each dot represents a repertoire. The y-axis represents the average frequency of a TCR in a repertoire. It is clear that there is not a big difference in the frequency distribution between positive and negative repertoires. [file Image_1.jpeg]

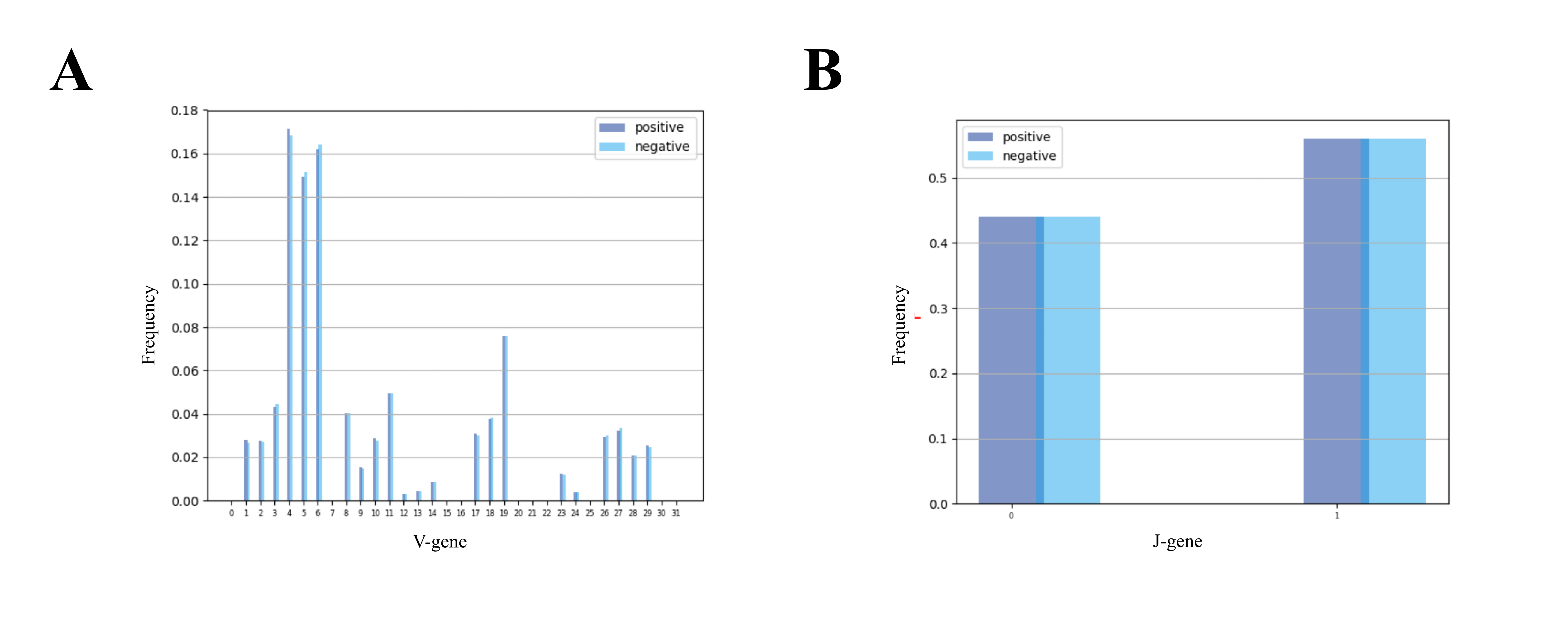

Supplement: Supplementary Image 2 — (A) A histogram of the different Vb-genes in the data. Each column represents the average frequency of a Vb-gene in positive and negative repertoires. It is clear that the v-gene distribution between negative and positive repertoires is very similar. (B) A histogram of the different Jb-genes in the data. Each column represents the average frequency of a Jb-gene in positive and negative repertoires. It is clear that the J-gene distribution between negative and positive repertoires is very similar. [file Image_2.jpeg]
